# Improving Trapping Efficiency for Control of American Mink (*Neovison vison*) in Patagonia

**DOI:** 10.3390/ani12020142

**Published:** 2022-01-07

**Authors:** Gonzalo Medina-Vogel, Francisco Muñoz, Meredith Moeggenberg, Carlos Calvo-Mac, Macarena Barros-Lama, Nickolas Ulloa, Daniel J. Pons, B. Kay Clapperton

**Affiliations:** 1 Centro de Investigación para la Sustentabilidad, Facultad Ciencias de la Vida, Universidad Andrés Bello, República 440, Santiago 8320000, Chile; mandm@moeggenberg.org (M.M.); calo.25388@gmail.com (C.C.-M.); m.maca.barros@gmail.com (M.B.-L.); 2 Instituto de Bioquímica, Facultad de Ciencias, Universidad Austral de Chile, Isla Teja s/n, Valdivia 5090000, Chile; franciscoldx@gmail.com; 3 Departamento de Ecología, Facultad de Ciencias Biológicas, Pontificia Universidad Católica de Chile, Av. Libertador Bernardo O’Higgins 340, Santiago 8331150, Chile; niulloa@uc.cl; 4 Departamento de Matemáticas, Universidad Andrés Bello, República 298, Santiago 8370035, Chile; dpons@unab.cl; 5 Independent Researcher, Havelock North 4130, New Zealand; clapperton.lo@xtra.co.nz

**Keywords:** invasive species, predator control, conservation, scent, trapping, mustelid, management strategies, cost-benefit analyses, pest management, population management

## Abstract

**Simple Summary:**

Two main challenges to control invasive mammals are to maximize campaign efficacy and cost-effectiveness, and to avoid trapping other species. We designed and tested new protocols to address those challenges to control alien American mink in southern Chile. We recommend the use of the GMV-13 variant of collapsible wire cage with scent gland lure, as they are smaller, cheaper, easier to transport and effective at catching female mink and reducing the possibility of trapping native species. Trapping campaigns using GMV-13 should be conducted principally during summer, with a 200-m trap spacing, for up to 6 days before moving traps to a new site, with a combination of three days with female scent gland lure followed by three days with male scent gland lure. Our modelling reveals that this should result in the removal of at least 70% of the local estimated discrete mink population within the area covered by each transect.

**Abstract:**

Two main challenges when controlling alien American mink (*Neovison vison*) in Patagonia are to maximize campaign efficacy and cost-effectiveness and to avoid trapping native species. We designed and tested new variants of collapsible wire box traps, compared the efficacy of a food-based bait and a scent lure and compared catch rates in different seasons of the year. We used the data to model the efficiency rate of the trapping and to determine the trapping effort required to remove 70–90% of the estimated discrete mink population. Between January 2018 and March 2021, we operated 59 trapping transects over 103 three-day trapping periods in southern Chile. Traps were first baited with canned fish, and afterwards with mink anal gland lure. We compared the efficacy of mink capture with that of our previous study. We trapped 196 mink (125 males, 71 females), with most captures in summer. The medium-sized GMV-18 trap caught more male mink, but the more compact GMV-13 caught fewer non-target rodents and no native mammals. The scent lure was more successful than the canned fish when the previous campaign’s data were included in the analysis. There was also a significant improvement in the proportion of female mink trapped and reduced labour compared with our previous campaign that used larger traps, fish bait and 400–500 m trap spacings. We caught relatively more females than males after the third night of trapping on a transect. Our data analysis supports the use of the GMV-13 variant of wire cage trap as the best trap size: it is effective on female mink, small, cheap and easy to transport. Combined with mink anal scent lure, it reduces the possibility of trapping native species compared with other traps tested in Chile. As the most efficient method for removing at least 70% of the estimated discrete mink population within the area covered by each trap transect in southern Chile tested to date, we recommend trapping campaigns using GMV-13 during summer, with a 200-m trap spacing, for up to 6 days before moving traps to a new site, with a combination of three days with a female scent gland lure, followed by three days with a male scent gland lure.

## 1. Introduction

The American mink (*Neovison vison*) is a semi-aquatic carnivore native to North America, first introduced into Argentina and Chile in the 1930s for fur farming [1]. It was also introduced into Europe at the beginning of the 20th century with the same purpose [2]. Currently, due to its generalist feeding habit, high reproductive rate and mobility, and due to escapes and deliberate releases from fur farms, this species has established feral populations in Patagonia [3,4,5,6]. Nowadays mink populations in Chile are distributed between latitudes 38° and 55° S [7,8], and are continuing to spread [9].

The mink is recognized for its devastating impact on native wildlife, particularly predating ground nesting birds and small mammals [10,11,12,13,14,15,16,17,18]. Moreover, introduced mink can represent a health threat to endangered river otter (*Lontra provocax* Thomas, 1908) [4,19,20]. In the area currently invaded by American mink in southern Chile, there are several native carnivore species, some of them of conservation concern: including the pampas cat (*Leopardus colocolo*); Geoffroy’s cat (*Leopardus geoffroyi*); kodkod (*Leopardus guigna*); culpeo fox (*Lycalopex culpaeus*); Darwin fox (*Lycalopex fulvipes*); gray fox (*Lycalopex griseus*); lesser grison (*Galictis cuja*); patagonian lesser weasel (*Lyncodon patagonicus*); southern river otter; marine otter (*Lontra felina*); and two subspecies of skunks (*Conepatus chinga* and *C. chinga humboldti*) [21]. Large-scale control or eradication campaigns may have unwanted casualties when trapping individuals of these species, and thus we need more species- and site-specific methods that provide significant conservation benefits to Patagonian wildlife [16,22].

While much is known about mink behaviour and ecology in Britain and Europe [2,23,24,25,26,27] and various trapping protocols have been tested and established [28,29,30,31,32,33,34], there is scarce information available to determine best-practice control strategies for mink in Patagonia [11,35,36,37,38,39]. Thus, eradication of this widespread alien predator from mainland Patagonia is not likely to be cost-effective with currently available knowledge, techniques and funding [2,40,41]. Elimination of local populations in geographically discrete valleys may be possible if we can fine-tune our trapping techniques [9,42,43]. Successful mink elimination has been achieved from both islands and mainland areas in Britain [31,32,33,44].

To maximise trapping efficiency, individual mink must encounter a trap during their daily movements. While covering large areas with widely spaced traps can remove mobile males with large home ranges [45], it is likely to miss animals that travel less. Female minks usually have smaller home ranges than males [24,26,46,47,48]. The combination of reduced trappability of females due to their movement patterns with the fact that male mink mate with multiple females suggest that trapping activities aimed at population reduction should target females. While mink usually show the typical mustelid intrasexual territorial system of overlapping male and female home ranges [23,45,49,50,51], females can be spatially segregated from males, inhabiting small streams [26,52]. Movements can also be affected by season [24], with males moving more in the mating season [51,53] while females are likely to be less active then and during the period when they are raising a litter [54,55]. Both sexes can be less active in winter [56].

Trapping success is often higher for male mink than for females [22,41,57,58], especially in freshwater habitats [41]. We may be able to improve catch rates of females by increasing the density of traps. Some previous studies in Europe and in Patagonia have used trap spacings of 400–1000 m [31,33,41,59], and Roy et al. [32] assumed an effective trapping radius of 250 m. With average female home range estimates varying between 0.5 and 6 km of river length [47], it was assumed that these trapping regimes would result in at least one trap per female home range, allowing all individuals to encounter a trap during the trapping period. However, mink spend most of their time in a smaller core area of their home range [47,50], making it less likely for females to encounter a trap on any given night. Thus, trap spacing and days of campaigns must have an impact on the sex ratio of trapped mink. If traps are dispersed over a large area relative to home range size, those individuals with a smaller home range should be trapped less frequently (a-trap-density effect) or may not be trapped at all (an exclusion effect); in this case, male mink will always be trapped before, and in larger numbers than females. If we increase trap density, the probability of trapping females is predicted to increase because males are removed, and because more trap-nights are available for females. Both Melero et al. [47] and Medina-Vogel et al. [41] recommended reducing the distance between traps to 200 m to maximize the number of minks trapped.

Even if there are adequate traps set to cover the core areas of all individuals, some mink can avoid traps [49,52]. It is known that trap design, their settings, baits and trappers’ experience are variables that significantly affect results [41]. Both live trapping [30,31,32] and kill traps [22] have been used effectively, but kill trapping can pose a serious threat to non-target species unless placed on rafts in the waterways where non-aquatic species cannot access them [60]. Rafts are not practicable for the fast-flowing rivers and streams present in Chilean Patagonia. Live traps have the disadvantages of being bulky and heavy, so are difficult to transport. The wire cage traps typically used measure 14–24 cm wide/high by 45–80 cm long [22,31,33,36,41,43,49,61,62,63]. Those typically used in regional mink trapping in Patagonia weigh 2.2 kg. Smaller, lighter traps may allow for more traps to be deployed per unit effort/cost without losing efficacy in a control programme aimed at females, as American mink in southern Chile are lighter in weight than in in Europe [64,65,66], and females can be half the body size of males [24,67].

Another way to improve trapping efficiency may be to use attractive baits or lures. Food-based edible baits are attractive to mink but also attract non-target species [59]. Commercial mink scent lures have been available to fur-trappers for centuries [68], and mink are thought to be attracted to traps that have previously caught conspecifics [69]. While not all lures are effective [60,61], and mink can be caught in unbaited traps [58], natural product and synthetic lures based on mustelid scent gland extracts and body odours have been shown to be attractive to conspecifics [61,70,71,72,73,74,75]. They have also been shown to be attractive to closely related species [75,76]. Anal gland secretions have the advantages of being copious and easily removed from live or dead animals [72,73,77].

During 2018 in Chile, the Regional Government of the Los Lagos District assigned approximately US$560,000 to control mink in an area larger than 8394 km^2^; in 2015, a similar amount was assigned in the Los Ríos district, for an area of approximately 18,430 km^2^ [78]. To promote politicians and managers to make commitments for more ambitious management programmes to eventually consider eradication [34], we need to be able to quantify the effectiveness of our trapping improvements. Medina-Vogel et al. [41] modelled the relationship between the cumulative catch rate, the initial mink population and the probability of catching any individual mink. They considered the latter trapping probability to be dependent on factors that determine trapping efficacy (type of traps, attractant used, distance between traps, and time of year) and the trapping effort (trap-nights). Their model predicts that there is a decreasing capture rate as the trapping period (*t*) and the number of trap-nights (*F*(*t*)) increase, leading to an infinite time, with an infinite effort, to capture the whole population. It also allows for an assessment of the trapping efficiency rate, and a calculation of the proportion of the population removed.

In this study, we evaluate the numbers of male and female American mink and non-target species (rats, native and domestic mammals) trapped in two new variants of wire box traps, lured with either canned fish or mink scent, spaced 200 m apart in a series of river valleys and lakes in southern Chile in all four seasons of the year. We compare our results from our previous study [41] in the same area to determine whether these changes in trapping protocol affected trapping efficacy. We use the model developed by Medina-Vogel et al. [41] to determine the percentage of the estimated discrete mink population removed by the control campaign.

## 2. Materials and Methods

### 2.1. Study Area

**Figure 1:**
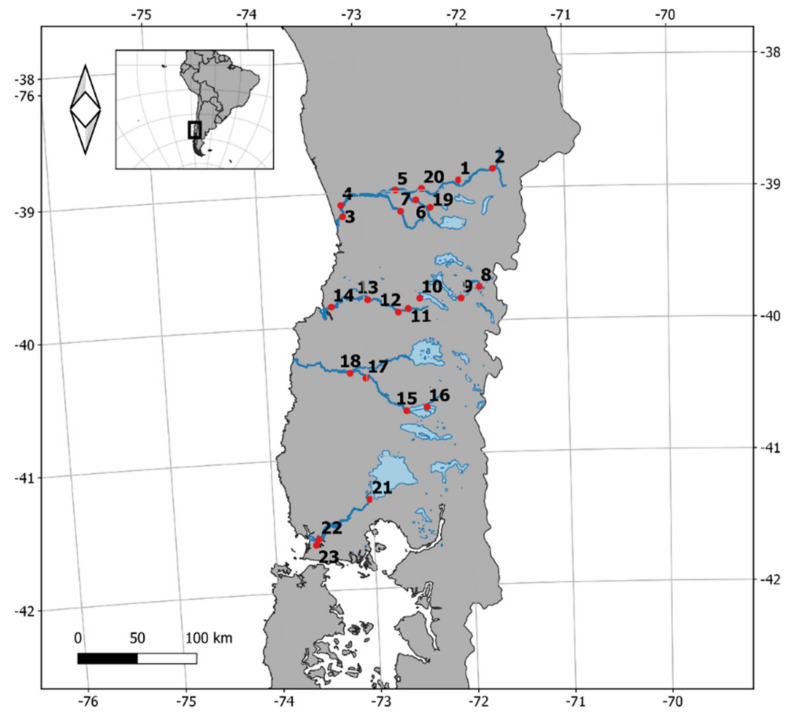
Geographic location of study sites within four river basins. 1, Cunco; 2, Melipeuco; 3, Nueva Toltén; 4, Hualpín; 5, Pitrufquén; 6, Coipué; 7, Donguil; 8, Liquiñe; 9, Choshuenco and Neltume lakes; 10, Riñihue; 11, Las Huellas; 12, Santa Rita; 13, Antilhue; 14, Valdivia; 15, Pilmaiquén; 16, Mantilhue; 17, San Pablo; 18, Trumao; 19, Catrico; 20, Radal; 21, Puerto Varas; 22, Maullín; and 23, González river.

This study was conducted in southern Chile between January 2018 and March 2021, between 39°0′24.5″ S to 40°40′4.8″ S latitude, and 71°33′36″ W to 73°49′53″ W (Figure 1). We visited 59 transects over 23 study sites in four river basins, separated either by a geographical barrier, or by more than 20 km following a river shore (Figure 1). The transects were likely thus to contain discrete mink populations [79]. The study sites included habitat beside rivers, streams and lakes but no marine habitats, and were within the same ecological region of our earlier campaign; in fact, we used three of the study sites (Neltume, Liquiñe and Maullín) during our 2009–2013 trapping [41,80]. These basins are in a region characterised by a temperate-humid-cool climate with 2000 to 3000 mm of rain a year, with an average humidity around 90%, and an average annual temperature below 10 °C [81,82].

### 2.2. Trapping

**Figure 2:**
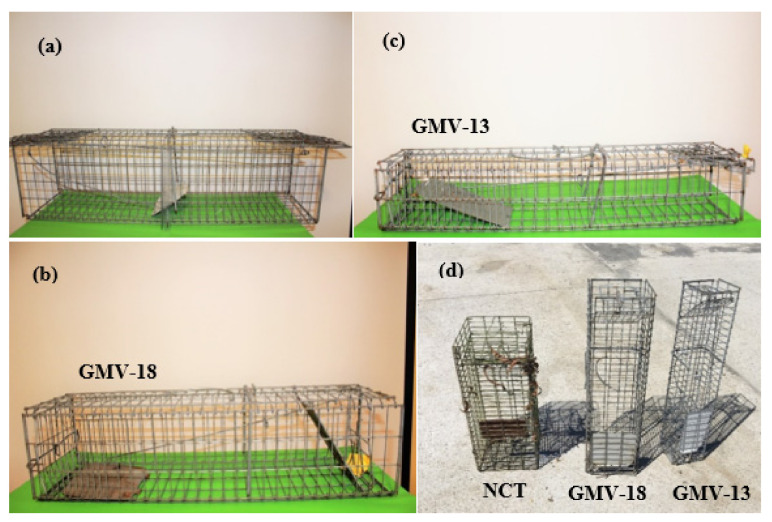
(**a**) Collapsible trap used in the 2010–2013 campaign (Medina- Vogel et al. [41]), (**b**) trap GMV-18 and (**c**) trap GMV-13, (**d**) comparison between GMV-18, GMV-13 and shorter non collapsible trap (NCT) used in Los Rios district, Chile by the regional program to control mink, showing the extended trap length and narrower spacing of bars in the trigger rear section of the GMV-13 and GMV-18, and the smaller width and height of the GMV-13. The GMV traps were non-commercial designs developed by our research team.

We trapped American mink along transects equipped with 5 to 42 collapsible single-entrance wire cage traps operated by one, two or three trappers. From January 2018 until August 2019, we used the GMV-18 trap (Figure 2), (18 cm high × 18 cm wide × 65 cm long, 1.6 kg); while from September 2019 until February 2020 and between January and March 2021, we used the GMV-13 trap (13 cm × 13 cm × 65 cm, 1.9 kg) (Figure 2). Both designs included a solid door, making it easy to see from a distance if they were sprung or not [83]. Depending on the study site characteristics and the existence of mink field signs such as scats and footprints, and on the experience of the local people, we set the traps at as close as practicable to a 200 m spacing. The transect lines followed the shore contour for 1 to 8.4 km on one side of the river (Table 1). Previous studies have shown the strong preference of mink for dense vegetation, steep riverbanks, behind rocks and under roots, in narrow passages, and more intensively in shallow rather than deep seashores [26,41,47]. We thus set the cage traps in places close to these features, and normally at no more than four metres away from the water. We also surveyed the covered sections for mink field signs to determine exact trap positioning.

We ran the transects for 2 to 10 days, with an average of six days, before being moved to new sites within the same study site. The sessions were divided into three-day periods for analysis. We rebaited the traps at the beginning of each session, or when the canned fish had been consumed. From January to June 2018 and in November 2019, the traps were baited with canned fish (Jurel en Tarro, San José^®^ Santiago, Chile). Throughout the rest of the study, the traps were baited with scent gland lure. We obtained the perianal scent bags from female or male mink trapped and killed during the current study, and stored them in glycerine. Before starting a new trapping session, these glands were opened, and the liquid was mixed with the glycerine. We mixed 5 mL of the scent in a Falcon tube with 4 mL of distilled water and 1 mL of 70% ethanol. We impregnated a cigarette filter with the solution, and tied it to the trap trigger in the same place as the canned fish bait previously used. Male or female or mixed-sex lures were randomly assigned to traps. All traps were checked each morning. We immobilized the captured mink humanely [84], mechanically with a plastic mesh and then chemically with an intramuscular (IM) administration of 10 mg/kg of Ketamine (Ketamina 100^®^, Chemie) and 0.025 mg/kg of Dexmedetomidine (Dexdomitor^®^, Pfizer). All mink were euthanized with thiopental (Tiopental Sódico^®^, Chemie).

We compare the results of these trapping sessions with those reported by Medina-Vogel et al. [41] and Barros et al. [80] in the Neltume, Liquiñe, Maullin, Cisnes river valleys, covering 4–15 km per day. These studies used 2.2 kg/21 cm × 23.5 cm × 81 cm cage traps with a double entrance (Figure 2), baited with fish bait, and spaced at 400–500 m.

### 2.3. Efficiency Rates and Estimation of the Proportion of the Population Removed

We use our trapping data as an input to test the model of trapping efficiency described by Medina-Vogel et al. [41] as

(1) $$X\left(t\right)=N\left(1\right)[\;1-\exp\{-kF(t)\}]$$

where *X*(*t*) denotes the cumulative number of animals trapped up to period *t*, and *F*(*t*) is the cumulative trapping effort (trap-nights) up to period t. With this data, we performed a non-linear regression using the Data Analysis application for iPad for each transect that was run for at least 6 days, using $X\left(t\right)$ and $F\left(t\right)$ for t = 1, 2, …, n, where n is the number of days at a given transect.

In order to quantify efficiency for comparison purposes, we obtain *N*(1), the estimated number (population) of individuals within the area covered by each trap transect at the beginning of the trapping session, and *k*, a measure of the efficiency of the trapping per trap: The value of *k* will be affected by the type of traps and the bait/scent used, by the distance between traps, and by the season and geography when/where the trapping takes place, as these factors affect the average speed of movement of the animal within its home range. Since *k* is very small, we can approximate 1-exp(−*k*) in Equation (1) with *k*.

We then determine the number of trap-nights (*F*(*m*) required to remove a certain percentage of the mink population, assuming a closed population over 6 days [41]; if we wish to remove the m% of the animals for a given value of k, we need at least the following trap-nights:

(2) $$\;F\left(m\right)=\frac{\ln\left\{\frac{100}{100-m}\right\}}{k}\;.$$

When the traps are set at a distance d from each other in a transect whose length is L, the number of days required to produce those trap-nights is calculated as:

(3) $$n=\frac{d}{Lk}\ln\left\{\frac{100}{100-m}\right\}.$$

### 2.4. Data Analysis

We evaluate the numbers of mink and non-target species (rats, native and domestic mammals) trapped per trap-night. We did not adjust the data for non-target captures or sprung traps [85] as our catch rates were very low, and the live traps were seldom sprung without a capture. We performed a stepwise regression to determine the relative impact of river basin, season, trap type and bait type on overall mink catch rates. The four seasons are defined as: Spring (21 September–21 December); Summer (21 December–21 March); Autumn (21 March–21 June) and Winter (21 June–21 September). To further compare catch rates on canned fish and scent lure, we included data from our previous trapping campaign in 2010–2013, as reported by Medina-Vogel et al. [41], and analyzed the results using General Linear Models in SYSTAT v. 12. Captures of non-target species were also analyzed using GLM. We assessed differences in male and female mink catch rates and between trapping periods using Mann-Whitney *U*. For all statistical tests, we set the significance at ≤0.05.

## 3. Results

### 3.1. Trapping

**Figure 3:**
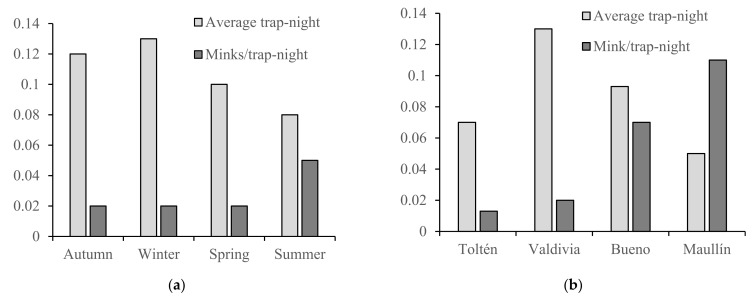
Average trap-nights per transect compared with average mink trapped per trap-night for (**a**) the four seasons and (**b**) the four study river basins.

**Figure 4:**
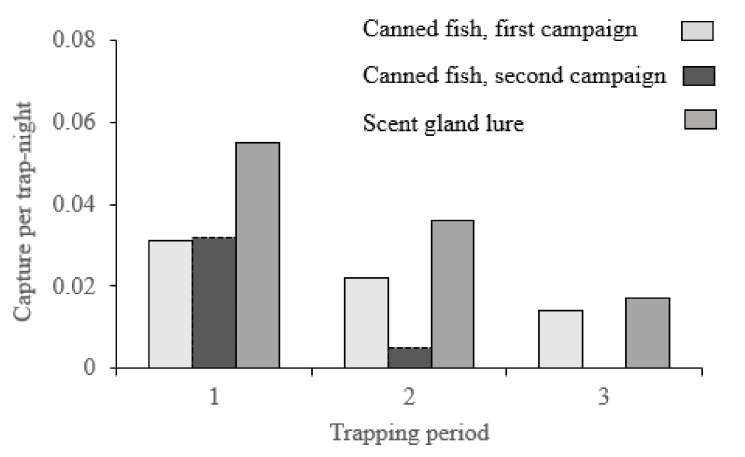
Comparative mean of mink trap-night using canned fish and scent lure over consecutive 3-day trapping periods (1–3; 4–6; and 7–9 days) during two trapping campaigns: 2009–2013 (data from the campaign reported by Medina-Vogel et al. [41]) and 2018–2021 (the current study).

We set a total of 6161 trap-nights over 103 periods of three days each and 59 trapping transects. We trapped 196 minks, 36% of which were females and 64% were males (Table 1). The two lake habitats returned lower catch rates than either the rivers or streams in the corresponding seasons (Table 1). Most mink were trapped in Summer (140), followed by Winter (25), Spring (17) and Autumn (14), with significant differences in mink per trap-night (*F*_3–99_ = 5.4; *p* < 0.01, Tukey post hoc test: *p* < 0.04 with 95% Confidence Interval −0.07/−0.006) (Table 1). Summer and Spring combined recorded a higher catch rate than Autumn/Winter for both males and females (Figure 3a). However, if we include the number of transects per season – Autumn and Winter (21% each), Spring (28%) and Summer (30%) in the analysis – the effort was more evenly distributed across the seasons, and trap-nights has no significant effect on total number of mink trapped (*F*_3–99_ = 1.0; *p* = 0.40). We spent fewer nights trapping per transect in summer but covered more transects, and this strategy provided the highest catch of mink (Figure 3a). This is probably because mink trapping rates dropped after the 6th day, a pattern that was also observed during the first campaign and was consistent over bait types (Figure 4). Similarly, among the different study river basins, Maullín river had the highest mink catch rate per trap-night (*F*_3–99_ = 9.9; *p* < 0.01 Tukey post hoc test: *p* < 0.01 with 95% Confidence Interval −0.1/-0.04) (Table 1) but the lowest average number of trap-nights per transect (Figure 3b).

Analysing summer data only, to avoid confounding seasonal effects, the GMV-18 traps had a significantly higher mink catch rate than the GMV-13 (*F*_1–56_ = 6.7; *p* = 0.01, 95% Confidence Interval −0.07/−0.01), but the difference was significant for males (*F*_1–30_ = 5.7; *p* = 0.02) and not for females (*F*_1–21_ = 3.1; *p* = 0.09). While catch rates of mink on scent lure averaged more than double those on canned fish in 2018–2021 (Table 2), there was no significant difference between the catch rates (*F*_1–56_ = 0.6; *p* = 0.43, 95% Confidence Interval −0.07/0.03). However, there was a significantly higher catch rate on scent lure when we included the trapping data from our 2009–2013 campaign (*F*_2–247_ = 11.7; *p* < 0.01) (Table 2).

The summer catch rates accounted for 85.9% of the female captures compared with 63.2% of the males (Table 1). There were no overall significant differences in mink capture rates on male or female scent lure (*F*_1–25_ = 0.09; *p* = 0.76). Neither the catch rate of male mink nor that of female mink varied significantly with lure type (male, female or mixed) (*F*_2–29_ = 0.17; *p* = 0.8; *F*_2–20_ = 1.0; *p* = 0.4). The higher average catch rate of females *(F*_1–19_ = 1.6; *p* = 0.21) on male lure *(F*_1–25_ = 0.09; *p* = 0.77) was not significant (Table 3). Overall, a larger proportion of females was trapped during days 4 to 6, compared with the first three days (Mann-Whitney *U* Test: 1.025; *df* = 1; *p* = 0.01) – a response that was not observed in males (Mann-Whitney *U* Test: 1.235; *df* = 1; *p* = 0.45).

We trapped significantly fewer rats and other non-target mammals using GMV-13 traps than GMV-18 traps (*F*_1–100_ = 5.4; *p* = 0.02). The mink scent lures used in the GMV-18 traps did not deter rats, but four times more cats were caught on canned fish than on scent lure (Table 4).

### 3.2. Efficiency Rates and Estimation of the Proportion of the Population Removed

In Table 5, the first three columns display the discrete Theoretical population obtained as the product of the density of minks (based on the literature) and the length of the transect (see Medina Vogel et al., [41]) in the 22 study transects. The discrete theoretical populations are compared with the minks that were effectively trapped in this campaign in the following three columns. The column labelled *N*(1) denotes the discrete estimated mink population, obtained using the trapping data and the non-linear regression of equation (1); the subsequent column provides the ratio between *N*(1) and the discrete Theoretical population. The values of the other parameter obtained from Equation (1), namely *k*, are then given, with *k* × 1000 ranging from 3.5 to 72.4.

With these values of *k* in Equation (2), we obtain the numbers of trap-nights (*F*(*m*)) needed to remove *m* = 70%, 80% and 90% of the discrete estimated mink population within each of the 22 transects. The average trapping effort required to remove 70%, 80% and 90% of the population is 92.8, 124 and 177.4 trap-nights, respectively. This converts to an average of 4.64, 6.2 and 8.87 trapping days, respectively, based on 200-m spacings between traps and an average transect length of 4 km using Equation (3).

## 4. Discussion

To improve American mink trapping cost effectiveness in Patagonia, we need to get out more traps per unit $, make the traps perform more effectively, identify the best time of year and also target females. While mink population densities and movements are likely to vary between years owing to food availability and climatic conditions [86,87] and trapping results varied amongst river basins, our two studies have spanned six years of data, so we consider it possible to make inferences from this research-by-management study. The results of our current campaign identify some means to improve trapping effort. We improved catch rates by using smaller, lighter traps, baited with mink anal gland lure rather than canned fish, set at 200-m trap spacing and moved to new transects after six nights. The speed with which we could cover transects, because of logistics and weather limitations, at least partially determined the differences in trapping rates amongst the seasons and river basins. Furthermore, captures of female mink increased between days four and six. These measures resulted in a 7.1% increase in captures per trap-night, allowing for fewer trap-nights per transect and fewer days of trapping needed to remove 70–90% of the population compared with our previous campaign along rivers, lakeshores and seashore within the same region as this study [41]. We removed three times the number of minks in approximately the same number of working days (363 versus 387).

Both the trap variants tested are much cheaper to buy, lighter and more portable but more rigid when set up than the trap used in our previous campaign. The smaller trap, the GMV-13 variant, allowed for more species-specific trapping, but caught fewer male mink in summer than the GMV-18. Both these trap variants thus have value, depending upon whether there is high value in reducing non-target bycatch and targeting female mink or whether the total mink catch rate is of higher concern.

We achieved the highest mink catch rates in summer and the lowest in winter; this is in line with the reported seasonal movement patterns of mink and with other trapping programmes. Roy et al. [32] found that the best trapping success was in the mating season (late winter/early spring) and dispersion (summer/early autumn). The success during the mating season may be because of high testosterone levels in male mink, making them less trap-shy [49]. Females move around with their young in late summer and early autumn [58], but do not venture far from the den during the breeding season [23]. Melero et al. [88] found that females were less mobile than males in winter and adults were less active than subadults. Timing of control may, however, be influenced by the ecology of the prey species the control operation is trying to protect [89].

Our overall sex ratio of 1.76:1 (males:females) is not surprising, seeing that female mustelids are considered to be elusive [57,58]. However, catch rates of males and females have varied greatly amongst countries and habitats, and over time. Roy et al. (2015) caught 60% females at the beginning of their campaign in Scotland, with catch rates of females increasing over time [32]. We also caught a significantly higher proportion of female mink in 2018–2021 than reported by Medina-Vogel et al. [41] for 2009–2013 (36% cf. 25%). By contrast in Argentina, Fasola and Roesler [22] had a 76% drop in the female catch rate from one year to the next, having started with a 1.2:1 ratio.

Our success in catching females can be put down in part to the use of mink anal gland scent lure, possibly in particular the male scent. Like in the Scottish Isles, we caught both male and female mink on scent lures at higher rates than on fish bait [74,82]. Mink scent lure has also been used with fish bait successfully on rafts fitted with kill traps in Patagonia [22]. The response of female mink to male and female scent lures requires more investigation. Other mustelids have been shown to respond to odours of the opposite sex [77,90,91].

Trap locations and spacing may also have an impact on female catch rates. Our reduced trap spacing of 200 m may have meant more females encountered traps compared with our earlier campaign. This spacing has been proven to be effective elsewhere [49], and was recommended by Melero et al. [47]. Zabala et al. [29] were able to target female mink by trapping in small streams in Spain, achieving catch sex ratios of almost 1:1. Based on this knowledge, Zuberogoitia et al. [43] used traps 100 m apart in preferred female habitat. Moore et al. [61] used ≤5 traps per km and Craik [58] used 10 per km.

The other noteworthy result in this study is the increased female: male ratio in captures after three days of trapping. As the male catch rate did not significantly reduce, this result cannot be fully explained by changes in female behaviour because of the absence of dominant males. The females may have become more trappable as neophobia decreased, or simply because as females are less mobile, and take longer to encounter the traps [92]. Roy et al. [32] also found that mink were more likely to be caught in the first few days of trapping, but did not show any difference between the sexes in their female-dominated sex ratios.

During our first campaign, the estimated cost of mink trapping per transect was approximately US$1000 at the end of the six days of field work, setting up traps every 400 to 500 m, and mostly using a boat to cover long distances [41]. During our second campaign, by reducing the trap size and its weight, we were able to set up transects of up to 8.4 km in length by walking along river and stream shores in less than two days, more than doubling the traps per night using similar labour; indeed, during the first campaign, 15 km were equipped with 124 to 373 trap-nights [41]. With the new protocol and traps, our average was 354 trap-nights with a maximum of 865 trap-nights, so we were more efficient. By spacing the traps every 400–500 m, and using canned fish as bait, Medina-Vogel et al. [41] were able to trap 79% of the theoretical mink population [41]. By spacing the traps every 200 m and baiting with scent gland lure, we caught mink at more than double the rate of Medina-Vogel et al. [41], and at close to 70% of the estimated theoretical mink population, in concordance with our effort (trap-nights). Our data support the model of Medina-Vogel et al. [41], showing that there will be a decreasing capture rate as the length of the trapping period and the cumulative trapping effort increase, leading to a need to stay for an infinite time, with an infinite effort, to capture all the population: these facts should be quantified to design realistic and affordable campaigns. The quotient between the mink trapped and calculated theoretical mink population (Table 5) was higher in this campaign than in the 2009–2013 campaign [41], showing that we were more efficient, especially for females. The variability amongst transects in the catch rates relative to theoretical population emphasizes the need to better understand the ecology and behaviour of mink in Patagonia, and use a tailor-made adaptive management approach [93] rather than relying just upon information sourced from other habitats and parts of the world. More research is needed on the home ranges, territoriality and trap evasion of mink, and how these interact with prey availability and other ecological factors that determine the variation in mink density amongst landscapes.

## 5. Conclusions

In this study, we have shown that minor changes in trap style, luring, positioning and moving rapidly from one transect to another can have significant effects on the ability to capture invasive mustelids and allow more focus on removing females. While we cannot determine the extent to which these factors individually affect trapping rates, we can recommend the use of a small portable trap, together with a low-cost species-specific lure, with transects averaging 4 km long checked every morning, and moving quickly between transects to improve efficiency for large-scale trapping programmes for mink in the environmental conditions of southern Chile. Such an approach will allow the removal of at least 70% of the estimated discrete within the trap transect cover area mink population, and help to prevent the capture of native carnivores of conservation concern.

## Figures and Tables

**Table 1:** Transect details and trapping results presented by river basin. TN = trap-nights.

River Basin	No. Transects per Study Site	Habitat	Season	Trap Type		Transect Length (km)		Trapped Mink			Days	TN ^A^	Mink/TN
				18	13			Male	Female	Total			
Toltén	1	Stream	Autumn	X		4.0		0	0	0	5	71	0
	3	River	Autumn	X		4.4		5	1	6	19	300	0.020
	3	River	Winter	X		6.0		1	0	1	17	329	0.003
	3	River	Spring	X	X	2.0		2	0	2	18	174	0.011
	5	River	Summer	X		2.2		2	3	5	20	188	0.027
Valdivia	1	River	Autumn	X		7.4		7	1	8	8	241	0.033
	4	Stream	Winter	X		6.8		5	1	6	21	590	0.010
	4	River	Winter	X		6.0		11	3	14	26	498	0.028
	2	Lake	Winter	X		6.8		1	0	1	11	328	0.003
	5	River	Spring	X		8.4		11	4	15	30	722	0.021
	9	River	Summer	X	X	6.8		21	8	29	56	865	0.034
	2	Lake	Summer	X		6.8		1	2	3	16	435	0.007
	1	Stream	Summer		X	3.0		4	5	9	6	90	0.100
	2	River	Summer		X	3.4		6	2	8	14	227	0.035
Bueno	2	River	Winter		X	4.0		3	0	3	12	204	0.015
	2	River	Spring		X	4.0		0	0	0	10	152	0
	5	River	Summer	X	X	5.0		29	26	55	27	487	0.113
Maullín	3	River	Summer	X		2.4		10	10	20	9	80	0.250
	2	River	Summer		X	3.0		6	5	11	12	180	0.061
Total	59							125	71	196	337	6161	

^A^ Because of field conditions in some transects not all traps were set from the first day.

**Table 2:** Mean (±CI) mink trapped per trap-night with canned fish or scent lure, in two different campaigns. The data from 2009–2013 come from the campaign reported by Medina-Vogel et al. [41]; the 2018–2021 campaign is the current study.

Year of Campaign	Bait Comparison		Mean	Captures per Trap-Night			No. 3-Day Trapping Periods
				Tukey’s Post Hoc Test *p*-Value	95% Confidence Interval		
					Lower	Upper	
2009–2013	Canned fish	Canned fish	0.01	0.99	−0.03	0.02	121
2018–2021	Canned fish	Scent Lure	0.01	0.00	−0.05	−0.02	26
2018–2021	Canned fish	Scent Lure	0.04	0.02	−0.06	−0.01	103

**Table 3:** Mean (±CI) male and female mink trapped per trap-night during summer associated with the gender of the scent gland lure.

Mink Trapped		Capture per Trap-Night	Tukey’s Post Hoc Test *p*-Value	95% Confidence Interval		No. 3- Day Trapping Periods
Gender	Scent lure	Mean		Lower	Upper	
Female	Female	0.03				16
	Male	0.07	0.16	−0.09	0.02	10
Male	Female	0.05	0.10	−0.04	0.04	11
	Male	0.03				12

**Table 4:** Number of individuals per species trapped over 6161 trap-nights (and catch rate per trap-nights) according to bait type and trap variant.

Trap Variant ^A^	GMV-18		GMV-13
Trap-nights	1632	3043	1486
Bait type Mammal species	Canned Fish	Scent Lure	Scent Lure
Mink (*Neovison vison*)	14 (0.0086)	118 (0.0387	64 (0.0431)
Rat (*Rattus norvegicus*)	1 (0.0006)	25 (0.0082)	1 (0.0007)
Cat (*Felis silvestris catus*)	12 (0.0074)	3 (0.0010)	0
Small dog (*Canis lupus familiaris*)	1 (0.0006)	0	0
Skunk (*Conepatus chinga*)	0	1 (0.0003)	0
Hare (*Lepus europaeus*)	0	3 (0.0010)	0

^A^ The two trap variants were the 18 cm high × 18 cm wide × 65 cm long, 1.6 kg GMV-18 and the 13 cm × 13 cm × 65 cm, 1.9 kg GMV-13.

**Table 5:** Comparison of the discrete estimated population with discrete theoretical population (see Medina-Vogel et al. [41] and effort needed to remove 70%. 80% and 90% of the population.

Transects	Theoretical Population ^A^			Proportion of Trapped Mink Related to Theoretical Population			Estimated Population Size	Proportion of *N*(1) ^B^ from Theoretical Population	Trap Efficiency ^C^	Number of Trap-Nights Needed to Remove a % of the Estimated Discrete Mink Population		
	Male	Female	Total	Male	Female	Total	*N*(1)		*k* × 1000	70	80	90
Nueva Tolten	1.3	1.9	3.2	2.4	0.5	1.3	4.0	1.3	52.1	23.1	30.9	44.2
Liquiñe	2.3	3.4	5.6	0.9	0	0.4	2.1	0.4	19	63.4	84.7	121.2
Liquiñe	2.0	3	5.0	2.5	0	1.0	9.7	1.9	4.64	259.5	346.9	496.2
Cua Cua	1.2	1.8	3.0	2.5	0	1.0	3.1	1.0	67.6	17.8	23.8	34.1
Liquiñe alto	0.4	0.6	1.0	5.0	0	2.0	2.6	2.6	58.8	20.5	27.4	39.2
Liquiñe alto	0.6	0.9	1.5	3.3	0	1.3	2.1	1.4	72.4	16.6	22.2	31.8
Santa Rita	2.5	3.7	6.1	2.8	0.3	1.3	15.6	2.5	2.8	424.3	567.2	811.5
Antilhue	1.6	2.4	4.0	1.3	1.3	1.3	5.4	1.3	16.2	74.3	99.3	142.1
Antilhue	0.9	1.4	2.3	2.1	0.7	1.3	3.2	1.4	41.9	28.7	38.4	55.0
Antilhue	2.8	4.2	7.0	1.1	0	0.4	3.1	0.4	13.9	86.6	115.8	165.7
Valdivia	1.2	1.8	3.0	0.8	0.6	0.7	2.1	0.7	41.2	29.2	39.1	55.9
Valdivia	1.1	1.6	2.7	2.8	1.9	2.3	6.0	2.3	28.5	42.2	56.5	80.8
Mantilhue	1.5	2.2	3.7	3.4	0	1.4	5.3	1.4	61.9	19.5	26.0	37.2
Pilmaiquén	1.3	2	3.3	5.3	6	5.7	27.3	8.2	14.3	84.2	112.5	161.0
Santa Rita	1.3	2	3.3	0	1.5	0.9	4.2	1.3	12.11	99.4	132.9	190.1
Valdivia	0.4	0.6	1.0	5.0	0	2.0	2.8	2.8	62.5	19.3	25.8	36.8
Valdivia	1.3	1.9	3.2	7.1	2.1	4.1	20.1	6.4	9.9	121.9	162.9	233.1
San Pablo	1.5	2.2	3.7	2.0	0	0.8	3.3	0.9	35.1	34.3	45.9	65.6
Trumao	1.2	1.8	3.0	5.8	1.7	3.3	13.9	4.6	14.7	81.9	109.5	156.6
Antilhue	1	1.5	2.5	4	3.3	3.6	12.5	5.0	51.4	23.4	31.3	44.8
Santa Rita	1.1	1.7	2.8	5.3	0	2.1	14.4	5.1	3.5	344.4	460.4	658.6
Maullin	1	1.5	2.5	5	3.3	4.0	15.4	6.2	9.55	126.1	168.5	241.1
Average	1.3	2.0	3.3	3.2	1.1	1.9	8.1	2.7	31.5	92.8	124.0	177.4

^A^ Theoretical population = theoretical density based on home range sizes × length of transect. ^B^ *N*(1) = the population at the beginning of trapping as extrapolated from the model. ^C^ *k* is the probability of capture of an individual per trap and is multiplied by 1000 to provide readable figures.

## Data Availability

The data that support this study is displayed in the tables in the bulk of this work, so are some pictures of the traps used.
